# Striking the balance with epistemic injustice in healthcare: the case of Chronic Fatigue Syndrome/Myalgic Encephalomyelitis

**DOI:** 10.1007/s11019-020-09945-4

**Published:** 2020-03-13

**Authors:** Eleanor Alexandra Byrne

**Affiliations:** Department of Philosophy, University of York, York, UK

**Keywords:** Epistemic injustice, Healthcare, Philosophy of medicine, Philosophy of psychiatry, Chronic Fatigue Syndrome/Myalgic Encephalomyelitis

## Abstract

Miranda Fricker’s influential concept of epistemic injustice (Oxford University Press, Oxford, 2007) has recently seen application to many areas of interest, with an increasing body of healthcare research using the concept of epistemic injustice in order to develop both general frameworks and accounts of specific medical conditions and patient groups. This paper illuminates tensions that arise between taking steps to protect against committing epistemic injustice in healthcare, and taking steps to understand the complexity of one’s predicament and treat it accordingly. Work on epistemic injustice is therefore at risk of obfuscating legitimate and potentially fruitful inquiry. This paper uses Chronic Fatigue Syndrome/Myalgic Encephalomyelitis as a case study, but I suggest that the key problems identified could apply to other cases within healthcare, such as those classed as Medically Unexplained Illnesses, Functional Neurological Disorders and Psychiatric Disorders. Future work on epistemic injustice in healthcare must recognise and attend to this tension to protect against unsatisfactory attempts to correct epistemic injustice.

## Introduction

This paper investigates tensions that can arise when the concept of epistemic injustice is applied to healthcare, where a delicate balance must be struck between attributing appropriate degrees of respect and epistemic authority to both patients and medical professionals. When attempting to address epistemic oppression, one need take care to avoid perpetuating it in the process (Dotson 2012). I suggest that a failure to strike this balance carefully risks obfuscating legitimate and potentially fruitful lines of inquiry, serving to both undermine the epistemic status of the dominant group and restrict epistemic resources that may benefit patients.

Epistemic injustice is now a vast area of philosophical study (see Kidd et al. 2017). I will not engage with all formulations of epistemic injustice, but instead focus on pathocentric epistemic injustices, that is, those that target ill persons (Kidd and Carel 2018). This paper focuses specifically on Chronic Fatigue Syndrome/Myalgic Encephalomyelitis (hereafter CFS/ME), a distressing illness which deserves careful research. CFS/ME has long been, and continues to be, poorly understood and much-disputed. Historically, comparisons have been made between CFS/ME and ‘neurasthenia’, a loosely defined psychopathological disorder first labelled as such in the nineteenth century, characterised by persistent fatigue, anxiety and depression. In the 1980s, CFS earned its classification as a non-psychopathological medical disorder, though resistance persisted, and the condition was dismissed by some as a form of neurotic hypochondria and disparagingly called ‘yuppie flu’. There is little consensus about the nature of the illness within the medical community, the general public and even patient groups. Much medical research has been done, and with many interesting results (see for example Esfandyarpour 2019; Kristiansen 2019). However, the data have been insufficiently significant and consistent to settle dispute.^1^Many are becoming increasingly committed to the view that what is currently understood as CFS/ME represents a cluster of different conditions, but that until research clarifies this, the label remains a catch-all term.

The symptomatology of CFS/ME is heterogeneous, but is typically characterised by disabling fatigue, lasting six months or longer, that is not eased with rest; post-exertional malaise (PEM); sleep problems; muscle and joint pain; headaches; cognitive problems; flu-like symptoms; feeling dizzy, sick, or having heart palpitations. Since no bio-marker or causal mechanism has been uncontroversially identified for CFS/ME, the status of the CFS/ME patient is unclear. This poses many epistemic challenges. Some have proposed that CFS/ME should be classified as a ‘functional syndrome’, that is, a syndrome that cannot be explained by identifiable disease (Mayou and Farmer 2002). However, this has also been met with resistance, since the term is sometimes associated with an implication of psychogenesis, the role of which in CFS/ME still remains much disputed. Others have proposed that CFS/ME ought to be classified as a neurological disorder that is meaningfully distinct from any psychiatric condition, and it is on this understanding that the label ‘Myalgic Encephalopathy/Encephalomyelitis’ (ME) is adopted. However, labelling of the disorder remains disputed by clinicians, researchers and patients. There is no uncontroversial label for the illness, but for current purposes, in-line with other contemporary research, I will use the label CFS/ME.

## Epistemic injustice in medicine and healthcare

At its broadest, an epistemic injustice occurs when someone is harmed specifically in their capacity as a knower. Testimonial injustice, a particular form of epistemic injustice, occurs when a speaker is unfairly attributed a lower level of credibility than they reasonably deserve, because of the speaker’s membership of a certain negatively stereotyped group.^2^ Such negatively stereotyped groups include sex/gender, race, and as is key in this paper, one’s status as an ‘ill person’. Where ill people are negatively stereotyped, the speaker’s testimony might be unfairly dismissed, excluded or seen as less valuable than it would otherwise have been if they were not ill. It is only by virtue of being a member of the stereotyped group that their testimony is received and acted upon differently. In other words, ‘a speaker suffers testimonial injustice just if prejudice on the hearer’s part causes him to give the speaker less credibility than he would otherwise have given’ (Fricker 2007).

Hermeneutic injustice, on the other hand, is a structural problem that arises as a result of a collective shortfall in conceptual resources. In other words, hermeneutical injustice arises when a group of people struggle to understand their experience because of some inadequacy in the resources that are required to understand them, resulting in a marginalisation of that group (Fricker, 2007, p. 153). Such hermeneutic resources might be considered inadequate if they simply do not exist, if the resources do exist but are inaccessible to those who would benefit from them, and if the resources do exist and are accessible to the vulnerable group but lack uptake by others^3^. Hermeneutic injustice does not only affect the marginalised group in question, but it also negatively affects the hermeneutically unjust group, since this lack in resources results in a collective inadequacy of understanding. Both parties can be said to suffer from cognitive disablement, but the victim is also cognitively *disadvantaged* because they are prevented from understanding their own experience (Fricker, 2007, p. 151). Fricker has argued that this cognitive disablement prevents the victim of the injustice from understanding ‘a patch of experience which it is strongly in her interests to understand, for without that understanding she is left troubled, confused, and isolated’ (ibid).

Kidd and Carel (2014) have written that Fricker’s framework can help us to understand and articulate some of the epistemic harms experienced by ill persons in the clinical encounter. They argue that while the patient-clinician relationship is not inevitably unjust, it is structured in a way that makes certain pathocentric epistemic injustices likely to occur (p. 530). The authors cite these epistemic injustices as one probable cause for patient dissatisfaction, since a disregard of the patient’s perspective on some matter relating to their own experience of illness is often detrimental to patient well-being, serving to undermine and deter further patient engagement with medical professionals (p. 531).

Kidd and Carel argue that ill persons are especially vulnerable to testimonial injustice because there is often a presumptive attribution of certain characteristics to ill persons that negatively affects the perceived credibility of their testimony, such as cognitive unreliability and emotional instability. Accordingly, patient testimonies are often dismissed as irrelevant, confused, too emotional or time-consuming (pp. 529–30). Where there is testimonial *justice* in healthcare, so they argue, patient testimonies would be recognised, actively sought out, and judged to be, at least in certain respects, epistemically authoritative (p. 532). The authors state that medical professionals have epistemic authority over some matters, but that the same applies to patients, yet the various structures of medical institutions are such that the epistemic authority of the patients is often not accommodated. Kidd and Carel acknowledge occasions whereby the medical professional would be right to exercise epistemic authority, but maintain that in such cases, the clinician can often be overly dismissive (p. 531).

They also argue that ill persons are especially vulnerable to hermeneutic injustice because experiences of illness are often difficult to understand and communicate due to inadequately developed or respected hermeneutic resources (p. 529). Where there is testimonial justice, the clinician will recognise that their failing to make sense of the patient’s experience is not due to any fault of the patient. An appropriately ‘just’ clinician, they suggest, might say ‘the fact that I don’t understand you isn’t your fault but mine … I am untrained in the kind of articulacy you are using, and this hermeneutical context does not provide me with those resources’ (p. 532). Where there is hermeneutic injustice, then, testimony is not dismissed or disbelieved outright, but there exists a conceptual impoverishment. In which case, there is a lack of epistemic resources belonging to a particular institution, or a lack of epistemic resources being appropriately employed, which prevents the patient’s articulation of their illness-experience from being acknowledged and/or shaping clinical practice. Consequently, patient’s attempts to articulate their experience are often not adequately recognised by medical professionals. In this respect, the two injustices are closely linked and work to sustain one another.

Kidd and Carel discuss CFS/ME as a case in which an unfairly low degree of credibility is often attributed to patients. They write that many physicians do not recognise CFS/ME, and instead consider it a psychiatric, as opposed to a somatic, illness (p. 532). Because of this contested nosological status, patient reports of distinctively bodily symptoms can be dismissed and explained away under different interpretations. Such an interpretation might be that the patient is suffering from a psychiatric disorder, or that in extreme cases where children are affected, that the children are suffering from abuse. This has been documented as being traumatic for some when the interpretation of the medical professionals, the group with epistemic authority, is in tension with the considerably more vulnerable patient’s own understanding of their illness.

Hence, Blease et al. (2016) have offered a complementary and fuller analysis of epistemic injustice in CFS/ME. The authors cash out both forms of epistemic injustice as follows, citing them as reasons for patient dissatisfaction. They write that when patients are victim to testimonial injustice, they experience implicit and explicit negative stereotyping, leading to the downgrading of patient reports on their condition (p. 551). A relevant negative stereotype about patients with CFS/ME might be an assumption that they have a particular kind of personality. For example, in a study by Raine et al. (2004), one GP described ‘a certain personality trait that is chronic fatigue waiting to happen’, while another GP stated preference for treating patients with Irritable Bowel Syndrome over patients with CFS/ME because such patients are not as ‘heartsinky’, a term used by GPs to describe patients who ‘exasperate, defeat and overwhelm their doctors by their behaviour’ (O’Dowd 1988). Another negative stereotype might be that patients who self-conceive as having CFS/ME are untrustworthy and therefore incredible. Patients are often denied credibility because of the doubts about the legitimacy of CFS/ME, which easily bleed into doubts about the patient. As Blease et al. have stated, ‘uncertainty about the condition translates into uncertainty about its sufferers’ (2016, p. 550).

Blease et al. (2016) also argue that hermeneutic injustice affects CFS/ME patients because the conceptual impoverishment about CFS/ME is responsible for the lack of framework within which patients and medical professionals can make sense of the condition. Resources to make sense of the patient’s experience may be absent, or contested, and consequently, the experiences of patients may not be recognised as pointing towards warranting a diagnosis of CFS/ME (p. 553). Patients may present similarly to patients with depression, for example, and a lack of conceptual resources for distinguishing CFS/ME from depression might shift doctors towards interpreting the patient’s experience in a particular way which may be at odds with the preferred interpretation of the patient.

Blease et al. (2016) write that testimonial injustice is sustained and also accompanied by hermeneutic injustice, since healthcare professionals (the epistemically and practically authoritative group) fail to provide the appropriate training about CFS/ME to trainee physicians. This, they argue, facilitates prejudiced deflations of patient credibility, and/or ‘an unfair lack of shared concepts with which to make mutual sense of the experience of the patient’ (p. 555). Accordingly, the authors have highlighted a concerning degree of scepticism amongst some trainee medical professionals about the ‘reality’ of CFS/ME (p. 554). Doubts about the reality of the condition can be understood as suspicions about whether or not what is understood as CFS/ME is deserving of a legitimate medical classification of its own. Thoughts along this line might be that the cluster of symptoms that is recognised as CFS/ME might be better attributed to other existing conditions, again, which tend to be psychiatric, namely depression. On this view, CFS/ME represents no meaningful ‘kind’ of its own. A study of student opinions by Stenhoff et al. (2015), found that a significant number of trainee physicians expressed negative attitudes about CFS/ME, understanding it as mere ‘tiredness’. Blease et al. (2016) identified these attitudes as involving negative stereotyping of patients, and argued that this group are at a high risk of perpetuating testimonial injustices with their future patients, thus exacerbating hermeneutical gaps (p. 555). This, they argue, highlights the need for medical training on CFS/ME and other medically unexplained conditions.

## Acknowledging conceptual impoverishment

I argue that there is a need for caution in attributing testimonial injustice to particular concerns expressed by medical professionals dealing with CFS/ME, since the fundamental concerns in question are quite plausibly often legitimate. Over-liberally explaining general practitioners’ behaviour in terms of negative stereotyping of patients, I suggest, risks failing to recognise the pervasiveness of the conceptual impoverishment of CFS/ME, thus potentially hermeneutically disabling patients. Ironically, such negative stereotyping of medical practitioners’ behaviour would amount to a form of epistemic injustice directed at medical practitioners themselves^4^. Chew-Graham et al. (2010) surveyed GPs’ attitudes towards making a diagnosis of CFS/ME, and the subsequent management of patients in primary care. Some GPs responded:

Some people like a label, some people like to know what’s causing their symptoms whether it’s the truth or not and some people are looking for a label to attach to their symptoms. (GP17).

Once you start labelling a patient if you’re not careful you might have a self-fulfilling prophecy. (GP15)

At a superficial level it’s empowering because it gives them control over their life and their work, but at a deeper level it prevents them from engaging fully with the existential conditions of their life which is what they can’t cope with. (GP18)

I try to avoid it because once you give them the label you’re actually setting them off on a track which will get them nowhere. (GP14)

(Chew-Graham et al. 2010)

Blease et al. (2016) reference the response from GP15 to demonstrate that some GPs believe that a diagnosis of CFS/ME is inherently problematic, and frame this to be indicative of negative stereotyping of patients and the related suspicions about the legitimacy of CFS/ME as a genuine illness classification. As well as documenting GPs’ reluctance to diagnose CFS/ME, Blease et al. (2016) also argue that the responses in the study show that some GPs queried the value of referral as unnecessary or even harmful. Some GPs have indeed commented on the value of referral:

Well, I don’t think there is anyone to refer to. The specialist clinic is a waste of time; they just hold their hands up, “what can we do? Why, what are they sending this to us for?” (GP14).

Ultimately, these GP transcripts are intended to illustrate the prevalence of testimonial injustice insofar as they are indicative of patient testimonies not being acted on decisively as a result of disbelief about the condition, and so of the patient. However, it is not clear that this is the only and/or most plausible way to interpret these responses. I suggest that to frame them as plain evidence of negative stereotyping is too quick, and fails to properly appreciate the epistemic predicament of CFS/ME. It is important to recognise that people with CFS/ME most certainly *are* vulnerable to negative stereotyping, and that this can have a negative effect on how their testimony is assessed. However, I claim that at least some of the GP responses which have and may be continued to be identified as indicative of testimonial injustice instead demonstrate sensitivity to legitimate and potentially fruitful paths of inquiry, rather than negative stereotyping. Recognising this highlights the need to acknowledge the heterogeneity of the condition, and scrutinise the epistemic and conceptual problems surrounding it.

Consider one particular claim of testimonial injustice, that is, that delays in diagnosis point towards reluctance to take patient complaints seriously (p. 553). This is a good example of a case in which to find a perpetrator of testimonial injustice in the doctor who is slow to diagnose CFS/ME may not be an appropriate explanation. Moreover, to make such claims of negative stereotyping here might function as an obstacle to acknowledging the extent of the relevant conceptual problems with CFS/ME. Since there is not yet any bio-marker nor any other reliable ‘test’ for CFS/ME, the condition is diagnosed by exclusion, meaning that the process of diagnosis will tend to take as long as it takes to rule out all relevant alternative diagnoses. This points to conceptual problems with CFS/ME which it would be a moral and professional failure to under-appreciate.

What about the related problem of reluctance to diagnose? In this case, it is not just that medical professionals are slow to identify CFS/ME as the appropriate diagnosis, but that they are in some sense resistant to offer the diagnosis. Here, the argument can be made that doctors are reluctant to take complaints expressed by a patient as suggestive of CFS/ME due to scepticism about whether or not it is a legitimate illness category. Doctors may therefore adopt the view that the patient’s complaints might be better understood as belonging to some other diagnosis. To take this as plain evidence for negative stereotyping is to make a similarly problematic jump, since it is not obvious that reluctance to diagnose necessarily indicates negative stereotyping. Reluctance to diagnose certainly could be a consequence of negative stereotyping. However, the GP responses quoted by Chew-Graham et al. (2010) are not sufficiently revealing to confirm that this is the most appropriate explanation. We ought to recognise other legitimate reasons for GPs’ reluctance to diagnose CFS/ME that need not have involved negative stereotyping. For instance, reluctance to diagnose CFS/ME, and reluctance to refer patients to a specialist centre, can also be explained by a lack of compelling evidence that such diagnosis and referral tends to be beneficial for the patient, considering the lack of effective treatment resources for patients and the controversy that surrounds existing labels and treatment options, as suggested above by GP14.

Reluctance to diagnose could also be indicative of fear of misdiagnosing arising from uncertainty about CFS/ME given its conceptual impoverishment, as another response from this study suggests:

If someone else saw them who I felt was a good physician and also came to the same diagnosis as me then I would feel more confident that we were right. (GP15).

Although being able to understand one’s experience through the lens of a diagnostic label can bring patients great relief, receiving a diagnosis of a chronic condition may also be a source of distress, confirming that one’s situation is radically life-changing and with uncertain chance of effective treatment and full recovery. The response given by GP15, taken by Blease et al. (2016, p. 553) to be indicative of reluctance to accept CFS/ME as a ‘real clinical syndrome’, could just as plausibly demonstrate sensitivity to how diagnosis can shape experience and influence self-interpretation and behaviour. There is a considerable body of literature dedicated to exploring such dynamics in many cases of illness. Consider Hacking’s discussion of ‘looping effects’, where he argues that people with autism, once classified, do not develop just as individuals but as a group, modifying their experience and behaviour (1999, p. 112). Similar claims have been made about mental disorders, which once assembled as meaningful objects of discourse and practice, can have causal influences upon the experience and behaviour of those classified within that group (Weinberg 1997, p. 217). It is not clear that being conscious that a diagnosis of CFS/ME may put patients at risk of a ‘self-fulfilling prophecy’ is indicative of negative stereotyping. Rather, it is consistent with a wealth of medical and philosophical literature that demonstrates the influence of diagnosis, thoughts and beliefs on experience and behaviour.

The aforementioned concern of GP17, that some patients want to know what is causing their symptoms ‘whether it’s the truth or not’, may demonstrate that a doctor is aware of the relief and liberation that a diagnosis can bring to a patient but also that since CFS/ME appears to have highly heterogeneous aetiology and phenomenology between patients, what is currently understood about CFS/ME does not offer a clear causal story. Some patients may desire a label, and benefit from it, even though receiving the label does not illuminate their condition’s cause.

Moreover, GP18, who expressed concerns about a diagnosis preventing patients from engaging in the ‘existential conditions’ of their condition, may be demonstrating sensitivity to the fact that crudely dualistic understandings of illness remain present in society, not excluding some medical professionals (see Deary 2005; Fuchs 2005; Kendler 2005).^5^On such a crude understanding of illness, a medical diagnosis of a ‘somatic’ condition may be considered something that can be understood and treated exclusively biomedically. Perhaps GP18 recognises that a diagnosis of a somatic condition may prevent patients with such understandings from being reflective in ways that might benefit them, which may be of importance considering the heterogeneity of CFS/ME. As GP18 writes, this diagnosis might offer some relief, but where psychosocial factors like early-life trauma or psychiatric illness could be considered relevant to the development or maintenance of their condition, a CFS/ME diagnosis for a patient with such a view of illness could prevent them from engaging with such aspects of their condition which it could be within their interests to engage with.^6^ Insufficiently critical dismissal of such concerns as cases of negative stereotyping and epistemic injustice is done at the risk of restricting potentially fruitful and legitimate inquiry. Rather, by expressing such concerns, GPs may be demonstrating sensitivity to what might hermeneutically able and/or disable patients.

I suggest that at least some identifications of testimonial injustice in the GP responses listed are too quick. In many such cases, one can understand the responses as expressions of explicit or implicit sensitivity to a range of legitimate concerns that it is a medical professionals’ duty to consider and factor into one’s professional judgement considering the conceptual problems with CFS/ME. Taking these descriptions to be clear evidence of negative stereotyping is suggestive of the idea that the relevant injustices can be prevented by simply confronting the attitudes of the medical professionals in question. It is problematic to attribute negative stereotyping to GPs who may be expressing concerns that require action of a different kind, namely more work committed to trying to address the conceptual problems with CFS/ME.

## The patient as ‘knower’

There are surely cases of testimonial injustice that remain. One such injustice would be a medical professional’s failure to show appreciation for the heterogeneity in aetiology and phenomenology of CFS/ME. Such a medical professional may disbelieve any patient testimony that diverges from that preferred explanation, or even fail to recognise other plausible alternative explanations. The medical professional may therefore fail to properly respect, listen to or engage with testimony offered by the patient. Consider a case where a patient with CFS/ME is identified as having previously suffered from depression, and the physician’s knowledge of this is then used to undermine and dismiss any testimony offered by the patient that indicates that they have been correctly diagnosed with CFS/ME as opposed to Major Depressive Disorder (MDD). This is a clear case of unfair and unwarranted negative stereotyping.

There is, however, reason to be concerned about whether the notion of testimonial injustice is the best notion to make sense of the kinds of problems that *prima facie* might seem like cases of epistemic injustice against patients with CFS/ME. Recall that to suffer testimonial injustice is to be wronged specifically in one’s capacity as a provider of information. Fricker (2007) claims that to be a giver of knowledge is a fundamental part of what makes a human life valuable, and so not being taken seriously as such is to have one’s human value undermined. In undermining one’s capacity for reason, one’s humanity is undermined:

The capacity to give knowledge to others is one side of that many-sided capacity so significant in human beings: namely, the capacity for reason. We are long familiar with the idea, played out by the history of philosophy in many variations, that our rationality is what lends humanity its distinctive value… in contexts of oppression the powerful will be sure to undermine the powerless in just that capacity, for it provides a direct route to undermining them in their very humanity. (Fricker 2007, p. 44)

This formulation of testimonial injustice, then, commits itself to the idea that to suffer testimonial injustice is to have one’s rationality unfairly undermined. Indeed, the work on testimonial injustice done by Blease et al. (2016) largely functions as an attempt to highlight and protect against the negative stereotyping of CFS/ME patients as being irrational and thus undermined. However, there are cases in which this formulation of testimonial injustice cannot adequately capture what prima facie seems unjust. Edward Harcourt and Kate Martin have argued that this notion of epistemic injustice needs to be either rejected or refashioned so as to accommodate children and people with mental illness so that epistemic injustices can be said to have been committed against agents who are not fully rational (Edward Harcourt 2019, Royal Institute of Philosophy Lecture, University of York). I think that they are quite right here, but moreover, that illnesses like CFS/ME are especially epistemically challenging in a related way. Due to the uncertainty surrounding CFS/ME, patients are certainly vulnerable to epistemic injustice, principally because it is especially difficult to test the veracity of their epistemic contribution. The testimony of a child or mentally ill adult may be difficult to navigate in clinical practice, but in the context of many other illnesses, biomedical tests can be highly informative. Consider hypothyroidism, typical symptoms of which include fatigue, muscle and joint pain, and depression. Patient testimony here may be difficult to navigate, and even more so when the testimony is from a child or mentally ill person. However, with hypothyroidism and many other illnesses, blood tests can be done that are highly informative, correlating with and therefore serving to legitimate the testimony of the patient. In the absence of such tests for CFS/ME, the testimony of children and mentally ill people with CFS/ME is especially epistemically challenging, and thus, children and mentally ill people with CFS/ME are especially vulnerable to epistemic injustice (see Carel and Györffy 2014).

First, consider how the case of mental illness is relevant. There is a large body of patient testimony and medical research suggesting that suffering from a distressing chronic illness like CFS/ME can cause depression (De Jean et al. 2013; Castro-Marrero et al. 2017). Additionally, both patient testimony and medical research has also suggested that there are some cases of CFS/ME in which mental illness, most notably depression, has played some aetiological role in their condition, and other research has shown that the association between the two is at least more complicated than a straightforward case of reactive/secondary depression where the psychological strain of living with a certain condition gives rise to depressive symptoms (Goodwin 2006; Steptoe 2006; Harvey and Wessely 2009). In both of these cases, the person suffering from severe depression, by virtue of being depressed, is likely to suffer from a diminished or otherwise compromised capacity for decision-making, memory, and reasoning, and so in this sense, the rationality of the person with depression may be considered impaired (see Ratcliffe 2015 p. 273). Research has shown that both patients with CFS/ME and MDD performed worse on cognitive tests than healthy controls, although cognitive deficits were generally more subtle in those with CFS/ME (Lawrie et al. 2000). It is not obvious that the cognitive deficits identified in CFS/ME patients in this study, such as inhibited verbal fluency and recall/recognition, impair first-person insight. However, the study excluded participants with comorbid depression and CFS/ME, rates of which are high (Harvey 2009). Matthew Ratcliffe has pointed out that people with depression can lack insight into their condition; for example, one might only retrospectively gain insight into how isolated or lonely they felt when depressed (2015, p. 36). Moreover, although this particular example might be more common in depression or psychiatric illness, it seems that patient testimony can be similarly unreliable in the context of many other ‘somatic’ illnesses (Roberts 2010). Psychiatrist Ronald Pies has suggested that accordingly, patient narratives ought to be viewed with respect, but not credulity (2013, p. 290). Respecting testimony requires that one assesses it sensitively and seriously, but there follows from this no requirement to believe that it is true. As he has also written here about depression in the bereaved:

[T]he patient’s own “theory of the case” may prove to be misleading or incomplete; eg, the patient may be unaware of, or ignoring, the presence of an underlying medical disorder; unresolved intrapsychic conflicts; or environmental stressors not related directly to recent bereavement (ibid. p. 289).

Second, children can also suffer from CFS/ME. Incidence rates are unclear, though research suggests that there are two age-groups with elevated prevalence, children and adolescents aged 10–19, and adults aged 30–39 (Bakken et al. 2014; Crawley 2018). Moreover, high rates of comorbidity with depression, with various causal explanations, are also identified in this population, with research suggesting roughly one third of children affected can also be identified as depressed (Bould et al. 2011, 2013; Taylor et al. 2017). On Fricker’s formulation, testimonial injustice is an ‘epistemic insult’ in that it involves the intellectual undermining of an epistemic agent (2007, p. 49). As in the case of adults with depression, in the case of a child with CFS/ME, and possibly a child with CFS/ME and depression, recognition of the child’s cognitive limits may be necessary to avoid bigger ethical and professional failures.

It is implausible to maintain that the medical professional who acknowledges the potential severity and pervasiveness of such a patient’s predicament commits an injustice by acting in accordance with their judgement to exercise caution with regards to assuming patient testimony to be authoritative and/or accurate. It may be in the best interests of the patient with depression, reactive or primary, for the medical professional to be mindful of possible impairments to their rationality and first-person insight. Failing to do so and act accordingly can do a disservice to the predicament of the patient. This raises concern about claims of testimonial injustice being committed against a CFS/ME patient with depression, or against a child with CFS/ME, when the requirement for being victim to testimonial injustice is that one’s rationality is undermined.

Blease et al. (2016) recognise and accept the justified level of epistemic privilege that medical professionals have as a result of their training, but emphasise the need for recognition of different forms of epistemic contribution that patients can make, namely knowledge about the lived experience of their condition (p. 552. Knowledge that can be gained by engaging with a patient’s lived experience is certainly valuable. However, as I have stated, it is not obviously true that the first-person perspective on one’s illness is reliably comprehensively privileged in any group—not just in rational adults. A medical professional may have justified reasons to doubt that the child or depressed adult with CFS/ME can reliably understand and communicate the nature of their lived experience of their illness, some aspects of which are inextricably bound up with values, beliefs and interpretations. Similarly, an adult, rational CFS/ME patient is likely to have certain insights into their condition and their experience of it that the medical professional could not have grasped independently. However, the same patient may struggle to make sense of these experiences and convey them accurately, something which could be facilitated by the interpretative skill and relevant expertise of a medical professional. Claims that certain groups have epistemic privilege cannot easily be made even with respect to one particular epistemic domain, in this case, of lived experience: it does not seem to be the case that group *x* can claim epistemic privilege over matters *p* and *q*, whereas group *y* can claim epistemic privilege over matters *r* and *s*, and that both groups should take the other as epistemic authority on such matters. Rather, it seems that both groups *x* and *y* can contribute, sometimes independently and sometimes collaboratively, over *p*, *q*,* r* and *s*, and that accordingly, epistemic insight into CFS/ME ought to be a deeply collaborative exercise.

## Conclusion

I have highlighted two problems with how the concept of testimonial injustice might be applied to healthcare, using the example of CFS/ME. Although a useful concept which has already aptly shown how CFS/ME patients are vulnerable to suffering from epistemic harm, I have resisted claims that identify testimonial injustices where the epistemic problem can be just as plausibly explained by the medical professional exercising appropriate medical sensitivity within the context of a vast conceptual impoverishment. I have also argued that there is a problem with the idea of the CFS/ME patient as epistemically authoritative over their first-person lived experience, since their status as ‘knower’, of even their first-person lived experience, is highly complex.

I finished by suggesting that the process of gaining epistemic insight into CFS/ME ought to be deeply collaborative. Some researchers have suggested that, in psychiatry and medicine more broadly, there ought to be more collaboration between all ‘stakeholder groups’ (Fulford et al. 2014, p. 113). There are certainly promising avenues for further careful research into what the epistemic contributions of patients can reveal about CFS/ME. In the absence of a biomedical breakthrough, collaboration between patients, researchers, clinicians and medical professionals may prove to be the most effective way to enrichen the conceptual understanding of CFS/ME, empowering both patients and medical professionals.

## Footnotes

1 There is also considerable controversy around treatment options and their effectiveness (see White et al. 2007; Geraghty and Adeniji 2019; Geraghty and Blease 2018).

2 Fricker later distinguished between *discriminatory* and *distributive* epistemic injustice (2013). David Coady has since compellingly argued that all forms of epistemic injustice can be understood and treated as *distributive injustice* (2017).

3 Thank you to Ian James Kidd for prompting clarification of this important distinction.

4 Thank you to Matthew Ratcliffe for raising this point.

5 Identifying this particular dualistic view of illness as problematic does not necessarily undermine the entire project of philosophical dualism in all of its forms; this deserves careful exploration elsewhere (see Maung 2019).

6 I thank an anonymous referee for the comment that some consider CFS/ME to be a socially constructed condition. Although the social dimensions of illness are undeniably important, this view is troublingly metaphysically loaded, endorsing a dichotomy of natural and social kinds in illness. My inclination is that endorsing such a dichotomy will not prove a constructive path to pursue here. See Hacking (1999) for more on natural and social kinds.
